# Research and Implementation of Indoor 3D Positioning Algorithm Based on LED Visible Light Communication and Corresponding Parameter Estimation

**DOI:** 10.1155/2022/2940558

**Published:** 2022-09-13

**Authors:** Yi Li

**Affiliations:** School of Information Engineering, Xi'an University, Xi'an, Shaanxi, China

## Abstract

In the era of mobile Internet, the application of various positioning-based location service systems is becoming more and more common. In addition, the traditional radio positioning system is limited in the use of special environments such as mines, hospitals, and gas stations, and long-term electromagnetic radiation can cause potential damage to the human body. Compared with the traditional wireless positioning technology, VLC-based positioning technology has a good application prospect in the field of indoor wireless positioning. Compared with traditional radio positioning technology, the use of VLC technology to achieve indoor positioning is different in that the system design and layout need to consider the basic needs of indoor lighting; that is, the layout of multiple visible light sources in the room should meet the minimum illumination requirements of any area of the room. Since the layout structure of the light source that only considers the lighting requirements or only considers the positioning accuracy requirements is not the same, in the design process of the indoor visible light wireless positioning system, it is necessary to consider the overall optimization layout of multiple indoor visible light sources under the conditions of lighting and positioning constraints. This paper mainly optimizes indoor positioning from the aspects of light source layout, reflected light intensity distribution, and noise model.

## 1. Introduction of Characteristic Positioning Methods

### 1.1. Visible Light Communication (VLC)

Visible light communication (VLC), also known as nm-wave communication, is a communication technology that uses the visible light spectrum of 380∼740 nm as an information carrier. Because LED is highly efficient and has the characteristics of long life and fast response, it is more suitable for wireless communication than other visible light sources.

Compared with the traditional radio frequency (RF) wireless communication, VLC has the following advantages:

- VLC has an unauthorized bandwidth of about 400 THz and abundant spectrum resources
- VLC is easy to implement, allowing just adding a microcontroller to build a network using existing LED devices
- VLC uses visible light as a carrier and does not cause electromagnetic interference to other electronic devices
- VLC links can easily set up high-speed communication links above 10 Gb/s

**Figure 1:**
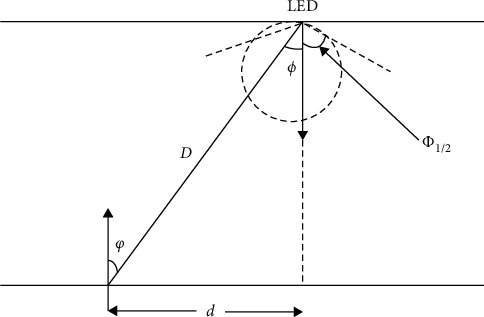
Lambert's model.

The RF, Bluetooth, and ultrasonic methods used in the indoor positioning system have problems such as low system stability, long response time, large electromagnetic interference, and low accuracy. The VLC is free from electromagnetic interference and can be achieved with fast and accurate positioning and navigation through the indoor fixed light source. In the future, large shopping malls, underground shopping centers, and other places can reduce the loss caused by customers being unable to find the specific location of the goods through the target positioning and guidance of the intelligent pedestrian automatic support system based on VLC. Similar applications can be extended to airports, museums, and other digital location locations and are also useful in the aerospace sector. Please refer to Figure 1.

The mathematical expression for the DC gain of the direct line-of-sight optical channel from the LED to the receiving end in the model is

(1) $$\begin{array}{c} H\left(0\right)=\left\{\begin{array}{c} \frac{\left(m+1\right)A}{2\pi D^{2}}{\text{cos}}^{m}\phi T_{s}gcos\varphi ,0\leq \varphi \leq \varphi_{e}\\ 0,\varphi >\varphi_{e} \end{array},\right. \end{array}$$

where *A* is the photodetector detection area, *D* is the distance between the transmitting end and the receiving end, *φ* is the angle of incidence, *ϕ* is the emission angle, *T*_*s*_ is the optical filter gain, *g* is the light concentrator gain, *φ*_*c*_ is the receiver perspective, and *m* is the light source radiation mode, and its mathematical expression is

(2) $$\begin{array}{c} m=\frac{\ln2}{lncos\Phi_{1/2}}, \end{array}$$

where Φ_1/2_ is the half angle of the luminous power of the light source; that is, the radiated power at this angle is half of the central power, and the *m* value size determines the beam directionality, and the greater the value, the better the beam directionality (1).

### 1.2. Maximum Likelihood Estimation Method

If the population *X* is of the discrete type, the form of its distribution law is known, which is the parameter to be estimated, and it is the range of possible values. Let it be a sample form; then, the joint distribution law is *P*{*X*=*x*}=*p*(*x*, *θ*), *θ* ∈ Θ*θ*Θ*θX*_1_, *X*_2_,…, *X*_*n*_*XX*_1_, *X*_2_,…, *X*_*n*_.

(3) $$\begin{array}{c} \overset{n}{\underset{i=1}{\prod }}p\left(x_{i},\theta \right). \end{array}$$

Also let *x*_1_, *x*_2_ …, *x*_*n*_ be a sample value of the corresponding sample. It is easy to know the probability that the sample will get the observed value; that is, the probability of the event occurring is *X*_1_, *X*_2_,…, *X*_*n*_*X*_1_, *X*_2_,…, *X*_*n*_*x*_1_, *x*_2_,…, *x*_*n*_{*X*_1_=*x*_1_, *X*_2_=*x*_2_,…*X*_*n*_=*x*_*n*_}.

(4) $$\begin{array}{c} L\left(\theta \right)=L\left(x_{1},x_{2}\ldots ,x_{n};\theta \right)=\overset{n}{\underset{i=1}{\prod }}p\left(x_{i};\theta \right),\theta \in \Theta . \end{array}$$

This probability *θ* varies with the value, and it is a function called the likelihood function of the sample (note that here are the known sample values, which are all constants): *θ*, *L*(*θ*)*x*_1_, *x*_2_ …, *x*_*n*_.

Regarding the maximum likelihood estimation method, we have the following intuitive idea: the sample value that is now known indicates that the probability of taking this sample value is relatively large, and we certainly do not consider the estimates of those that cannot make the sample appear; furthermore, if it is known that a large value is taken at that time and the other values are taken at a very small value, we naturally think that it is more reasonable to take the estimate as an unknown parameter. The maximum likelihood estimation method introduced by Fisher is to fix the sample observations and, within the possible range of values, select the parameter value that makes the likelihood function reach the maximum parameter value, and the most parameter estimate is taken as $x_{1},x_{2},\ldots ,x_{n}L\left(\theta \right)x_{1},x_{2}\ldots ,x_{n}\theta \in \Theta \theta \theta =\theta_{0}\in \Theta L\left(\theta \right)\Theta \theta L\left(\theta \right)\theta_{0}\theta x_{1},x_{2},\ldots ,x_{n}\theta \Theta L\left(x_{1},x_{2}\ldots ,x_{n};\theta \right)\tilde{\theta }\theta \tilde{\theta }$.

(5) $$\begin{array}{c} L\left(x_{1},x_{2}\ldots ,x_{n};\tilde{\theta }\right)=MAX\left(L\left(x_{1},x_{2}\ldots ,x_{n};\theta \right)\right)\theta \in \Theta . \end{array}$$

The resulting $\tilde{\theta }$ sample value is often denoted as the maximum likelihood estimate called the parameter, and the corresponding statistic is called the maximum likelihood estimator of the parameter: $x_{1},x_{2}\ldots ,x_{n}\tilde{\theta }\left(x_{1},x_{2}\ldots ,x_{n}\right),\theta \tilde{\theta }\left(X_{1},X_{2},\ldots ,X_{n}\right)\theta$.

If the population *X* is of the continuous type, the form of the probability density is known as the parameter to be estimated, and it is the possible value range. If it is a sample form, the joint probability density is *f*(*x*; *θ*), *θ* ∈ Θ, *θ*Θ*θX*_1_, *X*_2_,…, , *X*_*n*_*XX*_1_, *X*_2_,…, *X*_*n*_.

(6) $$\begin{array}{c} \overset{n}{\underset{i=1}{\prod }}f\left(x_{i},\theta \right). \end{array}$$

If *x*_1_, *x*_2_ …, *x*_*n*_ is a sample value corresponding to the sample, the probability that the random point falls in the field of points (the dimensional cube with the edge lengths are respectively) is approximately *X*_1_, *X*_2_,…, *X*_*n*_(*X*_1_, *X*_2_,…, *X*_*n*_)(*x*_1_, *x*_2_ …, *x*_*n*_)*dx*_1_, *dx*_2_,…, *dx*_*n*_*n*.

(7) $$\begin{array}{c} \overset{n}{\underset{i=1}{\prod }}f\left(x_{i};\theta \right)\text{d}x_{i}. \end{array}$$

Its value *θ* varies with the value taken. As in the case of the discrete type, we take an estimate that maximizes the probability (7), but the factor does not change with it, so only the function $\theta \tilde{\theta }\prod_{i=1}^{n}dx_{i}\theta$ is considered.

(8) $$\begin{array}{c} L\left(\theta \right)=L\left(x_{1},x_{2}\ldots ,x_{n};\theta \right)=\overset{n}{\underset{i=1}{\prod }}f\left(x_{i},\theta \right). \end{array}$$

The maximum value, here called the likelihood function of the sample, if*L*(*θ*)

(9) $$\begin{array}{c} L\left(x_{1},x_{2}\ldots ,x_{n};\tilde{\theta }\right)=MAX\left(L\left(x_{1},x_{2}\ldots ,x_{n};\theta \right)\right)\theta \in \Theta . \end{array}$$

The $\tilde{\theta }\left(x_{1},x_{2}\ldots ,x_{n}\right)$ maximum likelihood estimate is called the maximum likelihood estimate $\theta \tilde{\theta }\left(X_{1},X_{2},\ldots ,X_{n}\right)\theta$.

Thus, the problem of determining the maximum likelihood estimator boils down to the problem of maximization in calculus.

In many *p*(*x*; *θ*) cases and with respect to the microvariance, this is often available from the equation $f\left(x;\theta \right)\theta \tilde{\theta }$.

(10) $$\begin{array}{c} \frac{d}{d\theta }L\left(\theta \right)=0. \end{array}$$

$\tilde{\theta }$ takes the extreme value at the same place, so the maximum likelihood estimate can also be derived from the equation *L*(*θ*)ln*L*(*θ*)*θθθ*.

(11) $$\begin{array}{c} \frac{d}{d\theta }\ln L\left(\theta \right)=0. \end{array}$$

It is often more convenient to solve from the latter equation called the logarithmic likelihood equation.

**Figure 2:**
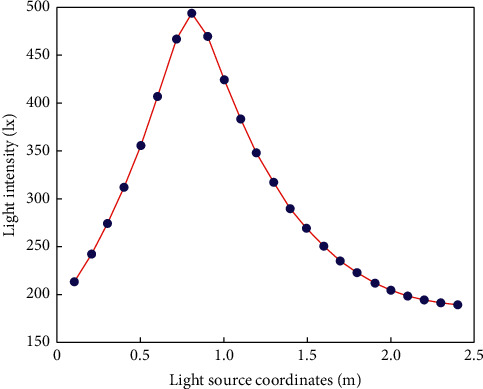
The minimum illuminance of different light positions.

In practical applications, the power of a single LED is small, usually using led array layout, in order to facilitate calculations; each LED array is regarded as a point light source, and the channel noise model is Gaussian white noise. The simulation uses the typical simulation environment shown in Figure 2, with four light sources symmetrically distributed on the ceiling board. Based on the Lambert channel model, the distance between each light source and the PD can be calculated using the formula [1], and then the trilateral positioning method can be applied [2].

Now I discuss the positioning of PD. Under the condition of indoor multilight source, the point light source signal received by PD may be greater than 3, and a system of super determinantal equations can be established, and the maximum likelihood estimation method can be used to estimate the PD position and further improve the positioning accuracy [3].

### 1.3. CRB Derivation in Indoor Environment

Indoor lighting requires conditions.

Illuminance represents the degree of light and darkness on the receiving surface and is defined as the luminous flux received per unit area in lux (IX). The illuminance on the receiving surface may be expressed as

(12) $$\begin{array}{c} E=\frac{I\left(0\right){\text{cos}}^{m}\phi }{D^{2}}. \end{array}$$

The luminous intensity *I*(0) of the LED center is used.

According to the relevant regulations of the International Organization for Standardization, the light intensity of ordinary indoors should generally be greater than 300 lx to meet indoor lighting requirements.

The location of the source installation will directly affect the illuminance distribution of the surface to be measured; here mainly consider whether the minimum illuminance of the surface to be measured meets the indoor lighting requirements (13), so the light source installation area that meets the indoor lighting requirements should first be calculated. The simulation parameters are shown in Table 1.

Because the four light sources are symmetrically distributed on the ceiling, their coordinate relationship is

(13) $$\begin{array}{c} \left\{\begin{array}{c} x_{1}=x_{2}\\ x_{3}=x_{4}=L-x_{1}\\ y_{1}=y_{3}\\ y_{2}=y_{4}=W-y_{1} \end{array}.\right. \end{array}$$

Due to its symmetry, when (*x*_1_, *y*_1_) is determined, the coordinates of the remaining three light sources are also determined, so only the position is discussed in this article (*x*_1_, *y*_1_).

(*x*_1_, *y*_1_)Move diagonally, that is*x*_1_ = *y*_1_, from the ceiling edge position (0.i, 0.i) to the near-ceiling center position (2.4, 2.4), in steps of

0.1, the remaining 3 lights move synchronously. Analyze the change trend of the minimum illuminance of the surface to be measured, and the simulation results are shown in Figure 2.

As can be seen from the figure, when light source 1 is installed in the *x*_1_=*y*_1_=0.4 and *x*_1_=*y*_1_=1.3 area, the minimum illuminance of the surface to be measured is greater than 300 lx, which meets the indoor illuminance requirements [4].

The CRB of the plane is received.

For the parameter estimation problem, the CRB boundary establishes a lower bound on the variance of any unbiased estimator; that is, it is impossible to find an unbiased estimator whose variance is less than the lower limit, and it provides a criterion for comparing the performances of unbiased estimates. In the study of this paper, the performance limit of the positioning error distribution of the coordinates of the point to be measured is CRB, so, by deriving the CRB of the point to be measured, the best performance of the positioning theory under the current environmental parameters can be obtained, which has a very important guiding significance for the layout of the visible light source array. The following is a theoretical derivation of the CRB of the test point [5].

According to the simulation environment and the Lambert model formula [6], the transmitted power of each light source is set to *P*_*ti*_, the received power corresponding to each light source at PD is *P*_*ri*_, and the distance from the light source to the PD is projected on the surface to be measured *d*_*i*_when the PD is placed horizontally: cos*φ*=cos*ϕ*. The noise distribution is Gaussian white noise *n*_*i*_, which can be obtained.

(14) $$\begin{array}{c} P_{ri}\left(d_{i}\right)=\frac{\left(m+1\right)A}{2\pi \left(d_{i}^{2}+h^{2}\right)}{\text{cos}}^{m+1}\phi_{i}T_{s}gP_{ti}+n_{i},i=1,2,3,4. \end{array}$$

Because $\cos\phi_{i}=h/\sqrt{d_{i}^{2}+h^{2}}$, one has

(15) $$\begin{array}{c} P_{ri}\left(d_{i}\right)=\frac{\left(m+1\right)A}{2\pi \left(d_{i}^{2}+h^{2}\right)}{\left(\frac{h}{\sqrt{d_{i}^{2}+h^{2}}}\right)}^{m+1}T_{s}gP_{ti}+n_{i}, \end{array}$$

(16) $$\begin{array}{c} f_{i}\left(d_{i}\right)=\frac{\left(m+1\right)A}{2\pi \left(d_{i}^{2}+h^{2}\right)}{\left(\frac{h}{\sqrt{d_{i}^{2}+h^{2}}}\right)}^{m+1},T_{s}gP_{ti}=\frac{\left(m+1\right)A}{2\pi {\left(d_{i}^{2}+h^{2}\right)}^{\left(m+3\right)/2}}h^{m+1}T_{s}gP_{ti}. \end{array}$$

Then *P*_*ri*_(*d*_*i*_)=*f*_*i*_(*d*_*i*_)+*n*_*i*_; let $f\left(d\right)=\left[\begin{array}{c} f_{1}\left(d_{1}\right)\\ f_{2}\left(d_{2}\right)\\ f_{3}\left(d_{3}\right)\\ f_{4}\left(d_{4}\right) \end{array}\right]$, and the vector $n=\left[\begin{array}{c} n_{1}\\ n_{2}\\ n_{3}\\ n_{4} \end{array}\right]$; then

(17) $$\begin{array}{c} \frac{\partial f\left(d\right)}{\partial d^{T}}=\left[\begin{array}{cccc} \frac{\partial f_{1}\left(d_{1}\right)}{\partial d_{1}} & \frac{\partial f_{1}\left(d_{1}\right)}{\partial d_{2}} & \frac{\partial f_{1}\left(d_{1}\right)}{\partial d_{3}} & \frac{\partial f_{1}\left(d_{1}\right)}{\partial d_{4}}\\ \frac{\partial f_{2}\left(d_{2}\right)}{\partial d_{1}} & \frac{\partial f_{2}\left(d_{2}\right)}{\partial d_{2}} & \frac{\partial f_{2}\left(d_{2}\right)}{\partial d_{3}} & \frac{\partial f_{2}\left(d_{2}\right)}{\partial d_{4}}\\ \frac{\partial f_{3}\left(d_{3}\right)}{\partial d_{1}} & \frac{\partial f_{3}\left(d_{3}\right)}{\partial d_{2}} & \frac{\partial f_{3}\left(d_{3}\right)}{\partial d_{3}} & \frac{\partial f_{3}\left(d_{3}\right)}{\partial d_{4}}\\ \frac{\partial f_{4}\left(d_{4}\right)}{\partial d_{1}} & \frac{\partial f_{4}\left(d_{4}\right)}{\partial d_{2}} & \frac{\partial f_{4}\left(d_{4}\right)}{\partial d_{4}} & \frac{\partial f_{4}\left(d_{4}\right)}{\partial d_{4}} \end{array}\frac{\partial f_{4}\left(d_{4}\right)}{\partial d_{1}}\right]. \end{array}$$

According to (15), *∂f*_*i*_(*d*_*i*_)/*∂d*_*j*_=0, *i* ≠ *j* and *∂f*_*i*_(*d*_*i*_)/*∂d*_*j*_=−*AT*_*s*_*gP*_*ti*_(*m*+1)(*m*+3)*h*^*m*+1^*d*_*i*_/2*π*(*d*_*i*_^2^+*h*^2^)^(*m*+5)/2^; therefore, *∂f*(*d*)/*∂d*^*T*^ diagonal matrix is

(18) $$\begin{array}{c} \frac{\partial f\left(d\right)}{\partial d^{T}}=-\frac{AT_{s}gh^{m+1}\left(m+1\right)\left(m+3\right)}{2\pi }\\ diag\left[\frac{P_{t1}d_{1}}{{\left(d_{1}^{2}+h^{2}\right)}^{m+5/2}},\frac{P_{t2}d_{2}}{{\left(d_{2}^{2}+h^{2}\right)}^{m+5/2}},\frac{P_{t3}d_{3}}{{\left(d_{3}^{2}+h^{2}\right)}^{\left(m+5\right)/2}},\frac{P_{t4}d_{4}}{{\left(d_{4}^{2}+h^{2}\right)}^{\left(m+5\right)/2}}\right]. \end{array}$$

On the surface to be measured, one has

(19) $$\begin{array}{c} \left\{\begin{array}{c} {\left(x_{1}-x\right)}^{2}+{\left(y_{1}-y\right)}^{2}=d_{1}^{2}\\ {\left(x_{2}-x\right)}^{2}+{\left(y_{2}-y\right)}^{2}=d_{2}^{2}\\ {\left(x_{3}-x\right)}^{2}+{\left(y_{3}-y\right)}^{2}=d_{3}^{2}\\ {\left(x_{4}-x\right)}^{2}+{\left(y_{4}-y\right)}^{2}=d_{4}^{2} \end{array}.\right. \end{array}$$

Set $X=\left[\begin{array}{c} x\\ y \end{array}\right]$; then one has

(20) $$\begin{array}{c} \frac{\partial d}{\partial X^{T}}=\left[\begin{array}{cc} \frac{\partial d_{1}}{\partial x} & \frac{\partial d_{1}}{\partial y}\\ \frac{\partial d_{2}}{\partial x} & \frac{\partial d_{2}}{\partial y}\\ \frac{\partial d_{3}}{\partial x} & \frac{\partial d_{3}}{\partial y}\\ \frac{\partial d_{4}}{\partial x} & \frac{\partial d_{4}}{\partial y} \end{array}\right]=\left[\begin{array}{cc} \frac{x-x_{1}}{d_{1}} & \frac{y-y_{1}}{d_{1}}\\ \frac{x-x_{2}}{d_{2}} & \frac{y-y_{2}}{d_{2}}\\ \frac{x-x_{3}}{d_{3}} & \frac{y-y_{3}}{d_{3}}\\ \frac{x-x_{4}}{d_{4}} & \frac{y-y_{1}}{d_{4}} \end{array}\right], \end{array}$$

According to the Cramero-Rao boundary formula, one has

(21) $$\begin{array}{c} {\left[B_{CR}\left(X\right)\right]}^{-1}={\left(\frac{\partial f\left(d\right)}{\partial X^{T}}\right)}^{T}Q^{-1}\frac{\partial f\left(d\right)}{\partial X^{T}}=F^{T}\left(X\right)Q^{-1}F\left(X\right), \end{array}$$

where *Q* is the covariance matrix of noise: *Q* = *E*[*nn*^*T*^]. According to the matrix differentiation formula, one has *∂d*(*d*)/*∂X*^*T*^ = *∂f*(*d*)/*∂X*^*T*^*∂d*/*∂X*^*T*^.

Combining formulas (18) and (20), one obtains

(22) $$\begin{array}{c} \frac{\partial f\left(d\right)}{\partial X^{T}}=\frac{\partial f\left(d\right)}{\partial d^{T}}\frac{\partial \left(d\right)}{\partial X^{T}}=-\left[\begin{array}{cc} \frac{P_{t1}\left(x-x_{1}\right)}{{\left(d_{1}^{2}\right)}^{\left(m+5\right)/2}} & \frac{P_{t1}\left(y-y_{1}\right)}{{\left(d_{1}^{2}+h^{2}\right)}^{\left(m+5\right)/2}}\\ \frac{P_{t2}\left(x-x_{2}\right)}{{\left(d_{2}^{2}+h^{2}\right)}^{\left(m+5\right)/2}} & \frac{P_{t2}\left(y-y_{2}\right)}{{\left(d_{2}^{2}+h^{2}\right)}^{\left(m+5\right)/2}}\\ \frac{P_{t3}\left(x-x_{3}\right)}{{\left(d_{3}^{2}+h^{2}\right)}^{\left(m+5\right)/2}} & \frac{P_{t3}\left(y-y_{3}\right)}{{\left(d_{3}^{2}+h^{2}\right)}^{\left(m+5\right)/2}}\\ \frac{P_{t4}\left(x-x_{4}\right)}{\left(P_{t4}\left(y-y_{4}\right)\right)} & \frac{{\left(d_{4}^{2}-h^{2}\right)}^{\left(m+5\right)/2}}{{\left(d_{4}^{2}+h^{2}\right)}^{\left(m+5\right)/2}} \end{array}\right], \end{array}$$

where *d*_*i*_(*i*=1, 2, 3, 4) can be obtained by formula (10). Replace formula (22) with formula (21) to obtain *B*_*CR*_(*X*).

### 1.4. Comprehensive and Best Performing Light Source Layout

**Figure 3:**
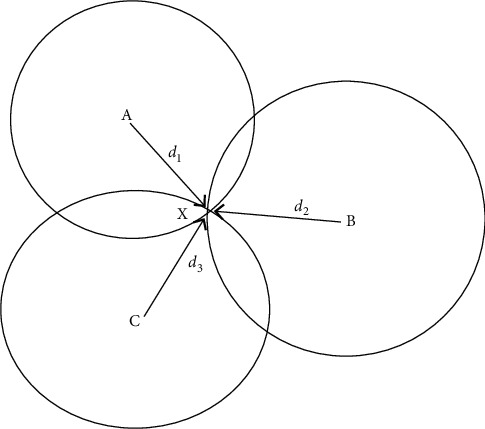
Schematic diagram of three sides measuring legal position.

According to the above derivation, for a given light source layout (i.e., given four light source coordinates), a CRB of any point to be measured can be obtained, and finally a CRB of the entire surface to be measured is obtained [7]. The lower the CRB, the higher the estimation accuracy that can be achieved. Therefore, it is hoped that, for the simulated indoor environment shown in Figure 2, under the premise of meeting the indoor lighting conditions, by adjusting the different light source positions [8], the CRB of the surface to be measured is as small as possible, and then the position of the light source corresponding to the smallest CRB is found, that is [9], the light source layout with the best positioning performance [10].

(*x*_1_, *y*_1_)Taking as an example, *x*_1_the sum *y*_1_is moved from 0.1 to 2.4 respectively , with a step size of 0.1, and the other three light sources move synchronously, and the signal-to-noise ratio at the receiving end is transmitted. The ratio is 30 dB, and the simulation results are shown in Figure 3.

## 2. MATLAB's Simulation of the Nearest Point Search Method

### 2.1. Matlab Simulation Positioning Scene

First of all, you need to build a 100 m*∗*100 m coordinate map, and the sampling point is 10 m in the map in step *M*. Take 5 points to be the AP point. Five actual position points were taken from the top, bottom, left, right, and middle of these 5 AP points. This is the result of the nearest point search method under the Matlab simulation [11], where the point represents the sample point taken, the five-pointed star represents the wireless access point, that is, the AP point, the difference indicates the actual location, and the point surrounded by the black triangle is the result of the nearest point search method.

### 2.2. Relationship between Step Length and Error

In general, as the step size decreases, the positioning will become more and more accurate, and the error will become smaller and smaller. In the nearest point search algorithm, the selection of the step size affects the size of the positioning result error; Table 1 is to select the coordinates of the 5 actual positions in the upper and lower left and right of the 5 AP points and then show the results and errors of their positioning under different steps. It specifically shows the results of positioning results and errors in different positions with the change of step size. As can be seen from the table, there is no error in positioning when the step size is 1, but the workload is too large when the step size is taken as 1, and it is impossible to imagine, so the step size of 1 is only an ideal value. According to several other zero errors, it can be concluded that there will be no errors only if the step size can be divisible by the horizontal ordinate coordinates of the actual position [12].

A plot of the step size and error of each point is given in the following. The relationship can be more directly observed through the graph.

**Figure 4:**
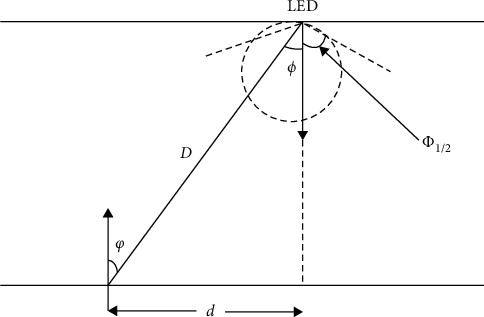
Schematic diagram of the emission of an LED light source.

As can be seen from the first 5 figures, the error generally shows an upward trend as the step size increases, and it can be seen that when the horizontal ordinate coordinates of the actual position are divided by the step length, the smaller the remainder, the more accurate the positioning, and in the cae of the integer division, the positioning is most accurate.  Figure 4 is a plot of five position steps versus error. This graph also reflects that the error generally increases with the increase in step length.

## 3. Channel Characteristics and Received Power Distribution

Use several criteria such as luminous flux, luminous intensity, emission power, and illuminance to measure the luminous capacity of LED light sources.

Luminous flux is the export of radiation flux evaluated according to the international standard human eye visual characteristics, which can be multiplied by the radiation energy of a certain band per unit of time and the relative visual rate of this band, even if the radiation power of different wavelengths of light is equal [13]; due to the visual rate of human beings for different wavelengths of light, the luminous flux is also different. Representation of the relationship between luminous flux and radiant flux is as follows:

(23) $$\begin{array}{c} \Phi =K_{m}\overset{780}{\underset{380}{\int }}V\left(\lambda \right)\Phi_{e}\left(\lambda \right)d\lambda . \end{array}$$

In the above formula, *K*_*m*_ is the maximum value of spectral photovisual performance, generally 6831 m/W, *V*(*λ*) indicates the standard spectral optometry efficiency function curve specified by the International Commission on Illumination (CIE), and Φ_*e*_(*λ*) indicates the spectral density of the radiation flux.

The emitted optical power represents the total energy of the light emitted by the light-emitting diode, expressed as follows:

(24) $$\begin{array}{c} P_{t}=\overset{\Lambda_{\text{max}}}{\underset{A_{\text{min}}}{\int }}\;\overset{2\pi }{\underset{0}{\int }}\Phi_{e}d\theta d\lambda , \end{array}$$

where Λ_max_ and *A*_min_ are the maximum and minimum values of the emitted light wavelength, determined by the sensitivity of the LED. The emission diagram of the LED light source is shown in (23).

Luminous intensity, also known as light intensity, indicates the brightness of the LED emitted light, and the unit is candela (cd); LED luminous intensity refers to the luminous flux emitted by the monochromatic light source in the unit stereo angle, which can be expressed as follows:

(25) $$\begin{array}{c} I=\frac{d\Phi }{d\Omega }, \end{array}$$

where the luminous flux is represented as Φ and the solid angle is represented as *Ω*.

As a reference point emitting light source, LED lamp is generally selected in line with Lambertian radiation model of the light source; its luminous model light intensity distribution is shown in Figure 4.

(26) $$\begin{array}{c} R\left(\theta \right)=\frac{\left(m+1\right)}{2\pi }{\text{cos}}^{m}\left(\theta \right)\theta \in \left[-\frac{\pi }{2},\frac{\pi }{2}\right]. \end{array}$$

In the above formula, the emission angle of the LED light source is represented by *θ*, and the *m* radiation order representing the directionality of the light beam can be calculated according to the emission half power angle [14].

(27) $$\begin{array}{c} m=-\frac{\ln2}{\ln\left(\cos\theta_{1/2}\right)}. \end{array}$$

### 3.1. Direct Model, the Emitter LED

The linear baseband signal transmission model of the visible light positioning communication system is shown in Figure 3.

In the direct model, the emitter LED *P*_*t*_ emits light with a modulated signal to the receiving end of the photometric PD, and the direct path channel gain between the two can be expressed as follows [14]:

(28) $$\begin{array}{c} H_{d}=\left\{\begin{array}{cc} \frac{\left(m+1\right)A_{R}}{2\pi d^{2}}{\text{cos}}^{m}\left(\theta \right)\cos\left(\psi \right)T_{S}\left(\psi \right)g\left(\psi \right) & 0\leq \theta \leq FOV,\\ 0 & \theta \geq FOV. \end{array}\right. \end{array}$$

In the above formula, *A*_*R*_ is the receiving area of the light detector, FOV is the field of view of the light receiver, *θ* is the radiation angle, *ψ* is the receiving angle, *T*_*S*_(*ψ*) is the gain of the receiving optical filter, and *g*(*ψ*) is the gain of the photopolymer, which can be expressed as follows, where the refractive index is represented by *γ* [15].

(29) $$\begin{array}{c} g\left(\psi \right)=\left\{\begin{array}{cc} \frac{\gamma^{2}}{{\text{sin}}^{2}\left(FOV\right)} & 0\leq \psi \leq FOV,\\ 0 & \psi \geq FOV. \end{array}\right. \end{array}$$

In the above model, the signal strength received by the receiving end PD from the transmitting source LED lamp can be described in signal power, so the strength of the received signal may be expressed as follows:

(30) $$\begin{array}{c} P_{r}=P_{t}*H_{d}. \end{array}$$

In theabove formula, the transmit power *P*_*t*_ represents the optical power, and, correspondingly, the received power *P*_*r*_ represents the received optical power, as noted herein *P*_*Opt*_. In the positioning system model, the receiving portion of the photodetector first converts the received optical power into the form of a current, and the conversion coefficient is *η*, represented by the following formula [16]:

(31) $$\begin{array}{c} i_{Elec}=\eta^{*}P_{Opt}. \end{array}$$

Then the corresponding received electrical signal power can be expressed as follows:

(32) $$\begin{array}{c} P_{Elec}=i_{Elec}^{2}R. \end{array}$$

This paper comprehensively considers the positioning of LED lamps as light sources for lighting, and the positioning system parameters based on MATLAB simulation are given in Table 2.

### 3.2. RSS Value Method for Ranging

In this article, the first use of RSS value method for ranging, for the Lambert order*m*, will be discussed in the following. In this simulation, we looked at the more common LED lamp data on the market, we can see that most of the LED lamps of the Lambert order is used*m* = 1. The distribution map of the intensity of the optical signal detected by the time detector at each point in the room emitted by the light source is shown in the MATLAB software simulation [17].

Regarding the reflection model, there are two more ideal models, which are specular reflection and complete diffuse reflection; the mirror reflection in the process of light in the transmission process touches a completely smooth nontransparent object and then reflects along the reflection angle exactly the same as the angle of incidence light; this process will form a very bright area, and the smoother the surface of the nontransparent object, the smaller the highlight area. Correspondingly, there is a special nontransparent object, generally made of high-purity technetium sulfate or magnesium oxide material; ideally its surface can absorb light from any direction and emit it in all directions, while the light intensity received in all directions is still the same [18]. The incident light under this fully diffuse mode is incident from one position, but the reflected light is emitted from any direction on the surface of the nontransparent object and can be evenly emitted. The above reflection model is a reflection of two extreme cases, and, in reality, the possibility of existing under the condition of ordinary latex paint wall or wooden floor and ceiling as reflective surfaces is not very likely. Therefore, in this paper, the reflection is simulated using the Phong lighting model, which is more classic in real graphics, not only considering the ideal specular and fully diffuse models but also combining the two in proportion. The ideal specular reflection is mainly to consider that the photon emitted by the LED lamp at the emitter end is incident on an ideal smooth plane at a certain angle and then reflected at the same angle as the angle of incidence, so the reflected light of the ideal smooth surface can only be seen in the direction of reflection. The full diffuse reflection model mainly considers that the photons are emitted from the emitting source and then transmitted again after receiving through the wall, and the photons are uniformly emitted in all directions with the wall receiving point as the center, so the reflected light can be observed at every angle near the wall [19].

Secondly, the photon emitted from the LED end, after a certain distance of attenuation, is received by a micrometer surface on the wall; at this time the attenuated power is expressed as follows:

(33) $$\begin{array}{c} P_{received}^{\left(0\right)}=H_{d}^{*}P_{source}^{\left(0\right)}. \end{array}$$

In the above formula, *H*_*d*_ is the gain in the DC channel in the upper section direct injection model, *P*_*source*_^(0)^ is the transmitted power of the LED lamp, and *P*_*received*_^(0)^ is the signal power received by the wall [20].

Then, each microelement surface on the wall can be regarded as a point light source in line with the Lambert model, and the signal power emitted outward needs to be absorbed by the wall in combination with the actual situation of the wall, so the power emitted outward can be expressed as follows:

(34) $$\begin{array}{c} P_{source}^{\left(1\right)}=\rho_{surface}^{*}P_{received}^{\left(0\right)}, \end{array}$$

where *ρ*_*surface*_ is the reflection coefficient caused by wall absorption.

Incident light is partially absorbed by the wall, and then, according to the Phong model, the ideal specular reflection *ρ* · *α* is achieved at the probability *ρ* · (1 − *α*), and the diffuse reflection is carried out at the probability, as shown in the following formula:

(35) $$\begin{array}{c} H_{r}=\frac{\rho \cdot \left(1-\alpha \right)}{\pi }\cdot \cos\left(\theta_{2}\right)+\frac{\rho \cdot \alpha \cdot \left(m+1\right)}{2\pi }\cdot {\text{cos}}^{m}\left(\theta_{2}-\psi_{1}\right). \end{array}$$

Then, after a reflection, the optical signal power received by the PD on the ground is

(36) $$\begin{array}{c} P_{received}^{\left(1\right)}=H_{r}^{*}P_{source}^{\left(1\right)}. \end{array}$$

### 3.3. Noise Model

In the visible light positioning system, the noise in the link has a great influence on the positioning accuracy, and the noise described in this section is the interference caused by other factors in removing the reflected light part of the wall received by the receiving end PD, which is defined as narrow noise [21].

In order to simplify the model, the noise is mainly considered to be a Gaussian noise model that obeys the mean value of zero and the variance, and the specific parameters are shown in Table 3. The power of the thermal noise is

(37) $$\begin{array}{c} \sigma_{TH}^{2}=\frac{8\pi kT_{e}}{G}\eta A_{R}I_{2}\beta^{2}+\frac{16\pi^{2}kT_{c}\Gamma }{g_{m}}\eta^{2}A_{R}^{2}I_{3}\beta^{3}. \end{array}$$

The power of the shot noise is expressed as follows:

(38) $$\begin{array}{c} \sigma_{shot}^{2}=2qRP_{BS}A_{R}\lambda \beta +2qRP_{r}\beta +2qI_{DC}\beta . \end{array}$$

### 3.4. Light Intensity Distribution of Reflection and Noise Models

This section gives a simulation of the distribution of reflected light and background noise other than reflected light in the indoor space; the power of the reflected light at the edge of the interior near the four walls is relatively small, but the power of the reflected light in the middle of the room is relatively small. There is direct radiation to the electrical signal intensity distribution chart; it can be seen that the maximum value of the signal intensity reaching the photo detector after a reflection is about 1/3 of the maximum value of the direct radiation reaching the signal intensity, so the impact of the reflected light on the positioning accuracy cannot be ignored, and, correspondingly [22], the intensity of the reflected light relative to the noise power after this reflection is used to achieve positioning under non-line-of-sight conditions. As can be seen from the signal power distribution of the reflected light such as the background light [23], since the shot noise dominates the noise model, it can be seen that the noise power and the power distribution in the direct beam case are in the same shape, and the noise power under the noise model is two orders of magnitude different from the direct signal power received in the line-of-sight link under the direct injection model, although there is no power of the reflected signal, but because the above is only to simplify the probability distribution assumed in the indoor environment given by the calculation, the distribution of the actual noise power is random, so the noise is still not negligible [24].

## 4. Visible Light Positioning Methods and Common Positioning Algorithms

### 4.1. Visible Light Indoor Positioning Method

The LED array located in a fixed position as a known reference point, LED in the illumination at the same time [25], will transmit out optical signal carrying the position information, as the unknown position of the receiving end of the optical detector for receiving the optical signal and detection and demodulation, while according to the received signal transmission time, angle of arrival or intensity, and then estimate the location of the specific moving target [26].

The positioning methods of LED indoor visible light positioning system are mainly divided into the following types: geometric measurement method, scenario analysis method, approximate perception method, and image sensor imaging method [27].

### 4.2. Geometric Surveys

The geometric measurement method is mainly on the two-dimensional plane, placing three LED lights on at least three known vertices as known position points and then estimating the distance between the LED at the transmitter end and that at the receiving end according to the time of receiving the signal [5], the signal strength, and the signal angle, and the commonly used positioning algorithms are RSS algorithm, TOA Algorithm, TDOA algorithm column, AOA algorithm, and negative and mixed localization algorithm [28].

#### 4.2.1. RSS Positioning Algorithm

RSS positioning is mainly by measuring the received signal strength to estimate the distance between the transceiver and sender ends, so as to estimate the location of the target point to be measured, which is a more common method of measuring the distance between the transceiver ends [29]. In the LED lamp-based visible light positioning system, when the position of the LED installed on the ceiling is determined, the LED is measured by emitting light with a signal, and the photodetector carried at the target position is moved to measure the intensity of the received light into a current intensity to measure the intensity of the light signal, using its own theoretical model to estimate the distance between the LED and the PD [30].

**Figure 5:**
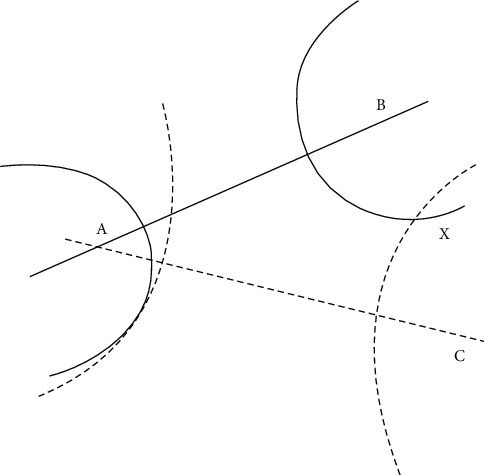
Three sides' position.

In the two-dimensional plane, at least three known position points are required as reference points, and these three known reference points are used as the center point, and the distance between the LED and PD obtained under the above received signal strength RSS attenuation is used as a half diameter to draw an arc; then the intersection of the three arcs is the estimated position of the target unknown point [31]. As shown in Figure 5, points A, B, and C are the positions of the three LED lights, *X* are the coordinate positions of the moving target points., According to the received signal strength, RSS measures the distance between the three LEDs and the PD, respectively is the sum of squares of *d*_1_, *d*_2_and *d*_3_ so that the intersection of the three circles is ideally the moving target position [11].

Based on RSS, the trilateral measurement of the value is mainly based on three known reference points A, Please refer to Figures 3 and 5.

Respectively(*x*_1_, *y*_1_),(*x*_2_, *y*_2_)和(*x*_3_, *y*_3_) can be used to find the coordinates of the moving target [32]:

(39) $$\begin{array}{c} \left\{\begin{array}{c} {\left(x-x_{1}\right)}^{2}+{\left(y-y_{1}\right)}^{2}=d_{1}^{2}\\ {\left(x-x_{2}\right)}^{2}+{\left(y-y_{2}\right)}^{2}=d_{2}^{2}\\ {\left(x-x_{3}\right)}^{2}+{\left(y-y_{3}\right)}^{2}=d_{3}^{2} \end{array}.\right. \end{array}$$

The above gives four common LED indoor visible light positioning basic algorithms and gives the comparison of these algorithms [33]; each algorithm can be positioned separately on the basis of sufficient reference points and measurement conditions, but it is often said in the real environment that some of these algorithms are mixed to achieve the purpose of amplifying advantages and reducing disadvantages. Compared to TOA and TDOA, the RSS and AOA methods achieve localization without full synchronization of time. Compared with RSS, TOA, and TDOA, AOA requires fewer reference points, but it requires relatively more hardware conditions and higher positioning costs [34]. Please refer to Table 4.

In order to build an indoor visible light communication system with considerable accuracy, this paper proposes an RSS positioning algorithm based on fingerprint data, which is a hybrid algorithm of RSS positioning algorithm and fingerprint library algorithm, to optimize the establishment of illumination model.

As described in equation (39), the optical power of order $d_{i}=\sqrt{d^{2}+r_{i}^{2}}$ can be expressed by the following equation:

(40) $$\begin{array}{c} P_{r}=\frac{\left(m+1\right)A}{2\pi d_{i}^{2}}{\text{cos}}^{m+1}\left(\theta \right)T_{s}\left(\theta \right)g\left(\theta \right)\times P_{t}. \end{array}$$

In the above formula, *A* is the photosensitive area, and *T*_*s*_ is the filter gain [12]. *g* is the light concentrating gain; it is the property of the photodetector itself; the transmitter power *P*_*t*_ is determined by the LED drive circuit and the LED itself; all of these parameters are unrelated to the external environment, LED layout, and other influencing factors, so the only vulnerable factor in equation (39) is the Lambert radiation coefficient *m*. In addition, in the physical sense, the Lambert radiation coefficient represents the directionality of luminescence, and its value is related to the half power angle, so if the illumination model does not match that shown in Figures 3–1, that is, the upper half power angles along different directions, the Lambert radiation coefficient *m* will also deviate. Repeated experimental measurements show that the LED contains more than one lamp bead, and the vertical distance between the LED and the light surface is not far in the order of magnitude greater than the lamp bead spacing. To sum up, how to accurately estimate the Lambert radiation coefficient *m* is the key to build the actual VLC indoor positioning system [35].

## 5. Conclusion

LED lamp green environmental protection pollution-free, high efficiency and energy saving safety, signal transmission speed, can provide considerable modulation bandwidth and other advantages. The LED light emitting system realizes the light emitting communication function and provides an opportunity for the rapid development of visible light communication technology. As the most important branch of visible light indoor communication applications, visible light positioning system has been concerned by many scholars and researchers at home and abroad because of its simple equipment and low system price [32].

In this paper, the reflection model of the wall surface is first proposed on the basis of the ideal direct beam model, and considering its impact on the indoor positioning effect, the Taylor series expansion iterative positioning algorithm and the classical least squares algorithm are used to achieve indoor positioning. On the basis of the original ideal classical least squares method, the improvement is proposed, taking into account the coefficients caused by the artificial arrangement of the transmitter [15]. Maximum likelihood estimation method is used to optimize the indoor positioning. RSS positioning algorithm and noise model are simultaneously mentioned in the article. Meanwhile, the author also provides detailed explanations regarding these problems. There are also errors in the matrix using the idea of the overall least squares to optimize the positioning algorithm, and finally the implementation of a variety of algorithms is compared and analyzed, and it is found that the new algorithm has achieved a relatively good positioning effect [36].

## Figures and Tables

**Table 1:** Errors of the coordinates' positioning under different steps of 5 actual positions.

Actual location	(88, 66)	(45, 55)	(52, 83)	(20, 50)	(49, 7)
Estimate position and error					
Step					
*M* = 1	(88, 66)	(45, 55)	(52, 83)	(20, 50)	(49, 7)
	0	0	0	0	0
*M* = 2	(88, 66)	(44, 54)	(52, 84)	(20, 50)	(48, 6)
	0	1.4142	1	0	1.4142
*M* = 3	(87, 66)	(45, 54)	(51, 84)	(21, 51)	(48, 6)
	1	1	1.4142	1.4142	1.4142
*M* = 4	(88, 64)	(44, 56)	(52, 84)	(20, 52)	(48, 8)
	2	1.4142	1	2	1.4142
*M* = 5	(90, 65)	(45, 55)	(50, 85)	(20, 50)	(50, 5)
	2.2361	0	2.8284	0	2.2361
*M* = 6	(90, 66)	(48 ,54)	(54, 84)	(18, 48)	(48, 6)
	2	3.1623	2.2361	2.8284	1.4142
*M* = 7	(91, 63)	(42, 56)	(49, 84)	(21, 49)	(49, 7)
	4.2426	3.1623	3.1623	1.4142	0
*M* = 8	(88, 64)	(48, 56)	(56, 80)	(24, 48)	(48, 8)
	2	3.1623	5	4.4721	1.4142
*M* = 9	(90, 63)	(45, 54)	(54, 81)	(18, 54)	(45,9)
	3.6056	1	2.8284	4.4721	4.4721
*M* = 10	(90, 70)	(50, 60)	(50, 80)	(20, 50)	(50, 10)
	4.4721	7.0711	3.6056	0	3.1623

**Table 2:** Positioning system parameters.

Parameter		Numeric value
Indoor environment	Reflection coefficient wall/ceiling/floor	0.66/0.35/0.66
	Room size	5.0 m × 5.0 m × 3.0 m
	Coefficient of refraction	1
Transmitter	LED coordinates	A[1, 1, 3]; B[4, 1, 3] C[1, 4, 3]; D[4, 4, 3]
	LED transmit power	1 W
	LED half power angle	60°
	Effective area	ldm × ldm
Receiver side	Sensitivity	0.4 A/W
	Receive the viewing angle	70°

**Table 3:** Positioning system parameters.

Symbol	Significance	Numeric value
*q*	Electronic charge	1.6 × 10^19^ C
*I*_*DC*_	Dark current	5 pA
Γ	Channel noise factor	1.5
*R*	Probe responsiveness	0.53 AAV
*β*	Equivalent noise bandwidth	400 MHz
*G*	Open-loop voltage gain	10
*K*	Boltzmann's constant	1.38 × 10^−23^
*T*_*e*_	Absolute temperature	300 K
*A*_*R*_	Detector effective area	1 dm^2^
*g*_*m*_	Transducer	30 m/s
*λ*	The bandwidth of the optical filter	400 nm
*P*_*BS*_	Background spectral irradiance	5.8 × 10^W/cm^2^
*η*	Fixed capacitance of photodetectors	112 pF/cm^2^

**Table 4:** Comparison of common LED indoor positioning algorithms.

Positioning algorithms	Targeting information	Merit	Shortcoming
RSS	Receive signal strength	No time synchronization required, simple, easy, and of low cost	The positioning accuracy is not high
TOA	Signal arrival time	High positioning accuracy	Between the transmit and receive ends, strict time synchronization is required
TDOA	Signal arrival time difference	High positioning accuracy	The time it takes to emit a light signal between the LEDs requires strict time synchronization
AOA	Signal reach angle	No time synchronization is required, positioning is simple in theory, and positioning can be achieved with fewer LEDs	A directional antenna is required, and the positioning cost is higher

## Data Availability

Data cannot be shared without permission.
